# Supplementary material for: Iterative improvement in the automatic modular design of robot swarms
Source: PeerJ Comput Sci. 2020 Dec 7;6:e322. doi: 10.7717/peerj-cs.322 (PMC7924708; doi:10.7717/peerj-cs.322)
Supplement: Supplemental Information 3 [file peerj-cs-06-322-s003.zip › argos3/doc/api/standalone/a00480_source.html]

ARGoS: plugins/robots/foot-bot/simulator/physx\_footbot\_model.cpp Source File


- Main Page
- Related Pages
- Namespaces
- Classes
- Files

- File List
- File Members

# plugins/robots/foot-bot/simulator/physx\_footbot\_model.cpp

Go to the documentation of this file.

```
00001 
00007 #include "physx_footbot_model.h"
00008 #include <argos3/plugins/robots/foot-bot/simulator/footbot_entity.h>
00009 
00010 namespace argos {
00011 
00012    /****************************************/
00013    /****************************************/
00014    
00015    CPhysXFootBotModel::CPhysXFootBotModel(CPhysXEngine& c_engine,
00016                                                       CFootBotEntity& c_entity) :
00017       CPhysXSingleBodyObjectModel(c_engine, c_entity),
00018       m_cFootBotEntity(c_entity) {
00019       /* Calculate base center */
00020       SetARGoSReferencePoint(physx::PxVec3(0.0f, 0.0f, 0.0f));
00021       /* Get position and orientation in this engine's representation */
00022       physx::PxVec3 cPos;
00023       CVector3ToPxVec3(GetEmbodiedEntity().GetOriginAnchor().Position, cPos);
00024       physx::PxQuat cOrient;
00025       CQuaternionToPxQuat(GetEmbodiedEntity().GetOriginAnchor().Orientation, cOrient);
00026       /* Create the transform
00027        * 1. a translation up by half body height
00028        * 2. a rotation around the base
00029        * 3. a translation to the final position
00030        */
00031       physx::PxTransform cTranslation1(physx::PxVec3(0.0f, 0.0f, 0.0f));
00032       physx::PxTransform cRotation(cOrient);
00033       physx::PxTransform cTranslation2(cPos);
00034       physx::PxTransform cFinalTrans = cTranslation2 * cRotation * cTranslation1;
00035       /* Calculate bounding box */
00036       CalculateBoundingBox();
00037    }
00038 
00039    /****************************************/
00040    /****************************************/
00041 
00042    void CPhysXFootBotModel::UpdateFromEntityStatus() {
00043    }
00044 
00045    /****************************************/
00046    /****************************************/
00047 
00048    REGISTER_STANDARD_PHYSX_OPERATIONS_ON_ENTITY(CFootBotEntity, CPhysXFootBotModel);
00049 
00050    /****************************************/
00051    /****************************************/
00052 
00053 }
```

---

Generated on 10 Jul 2018 for ARGoS by 
 1.6.1
